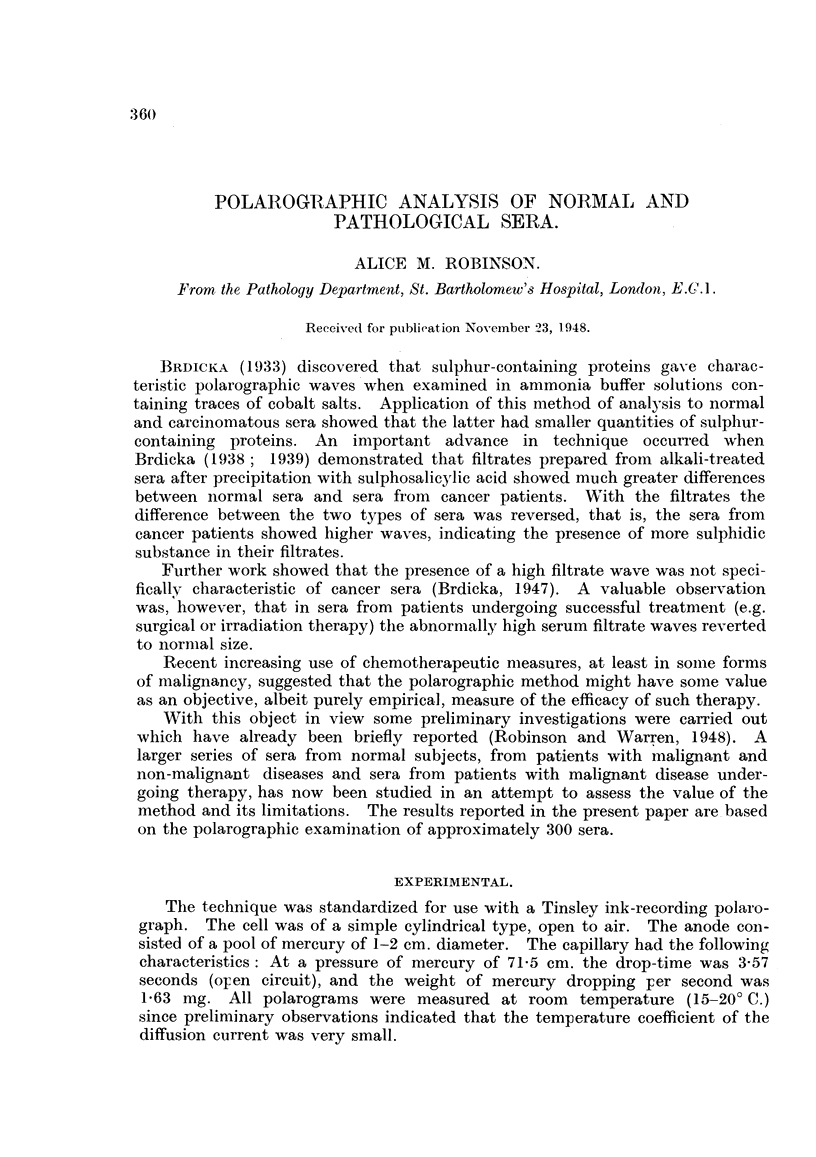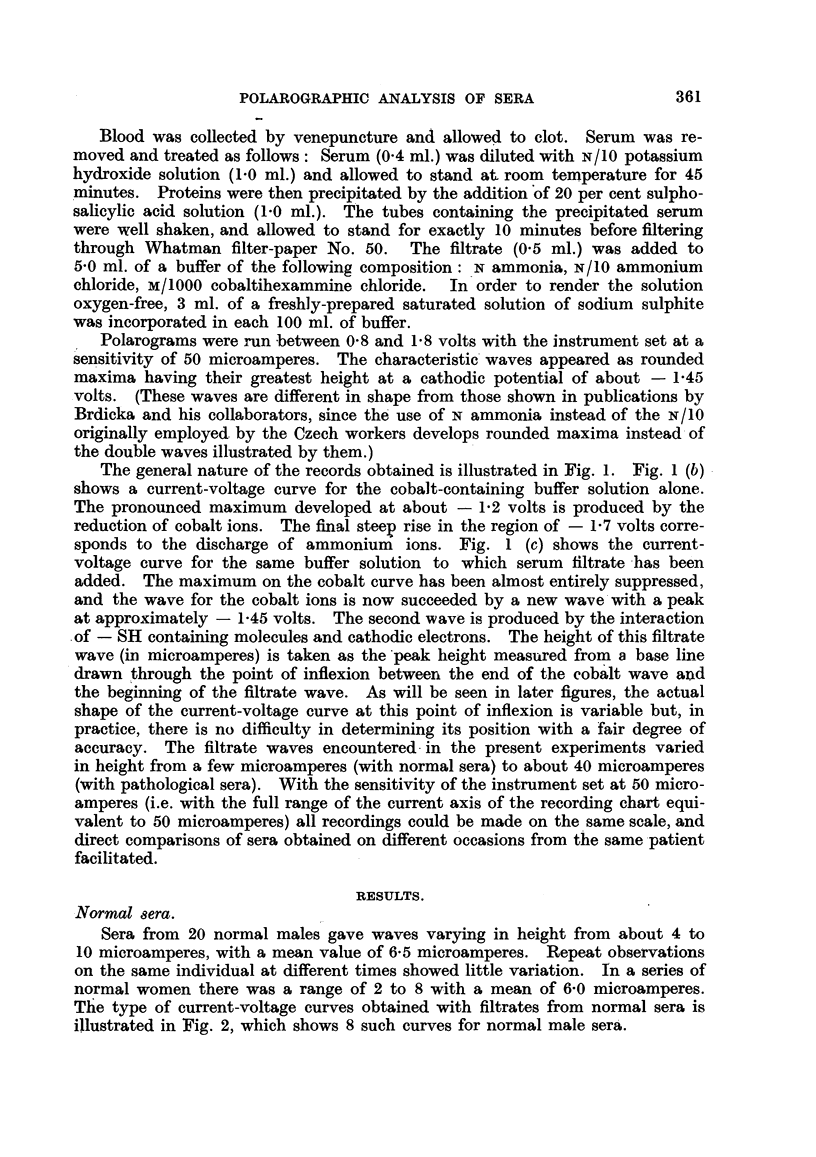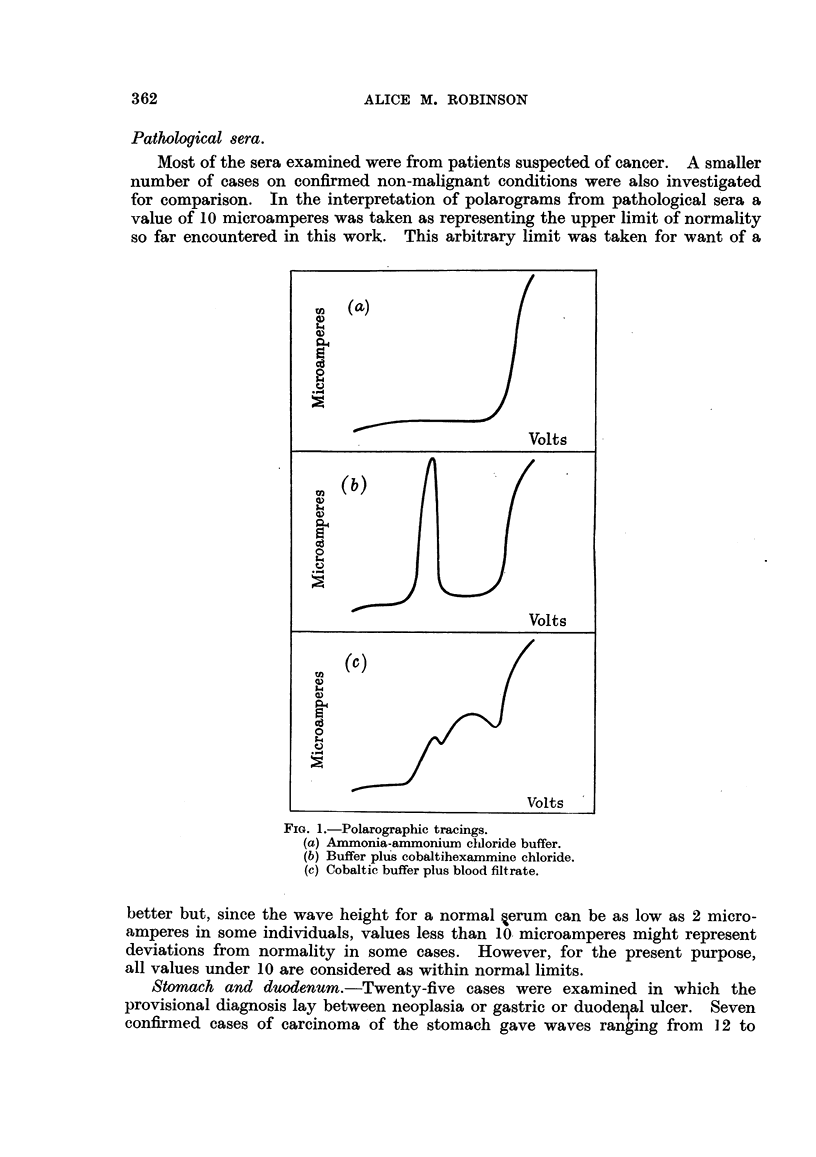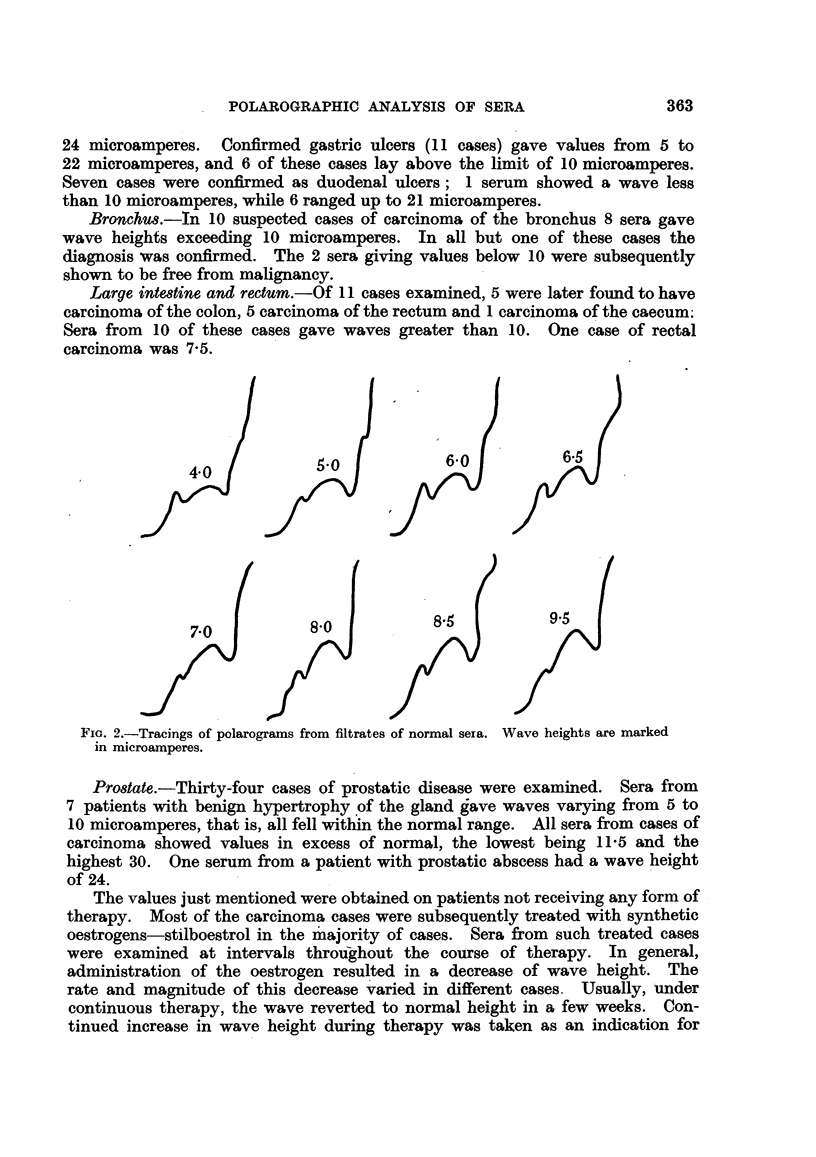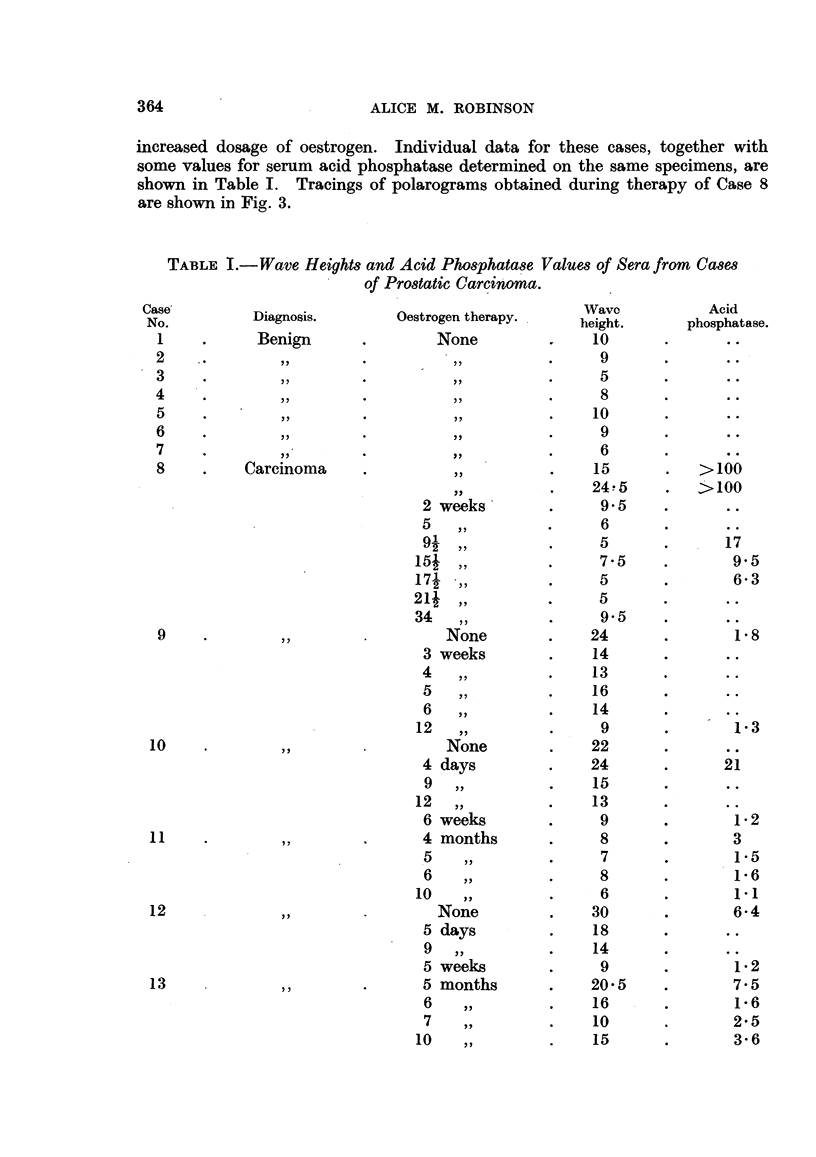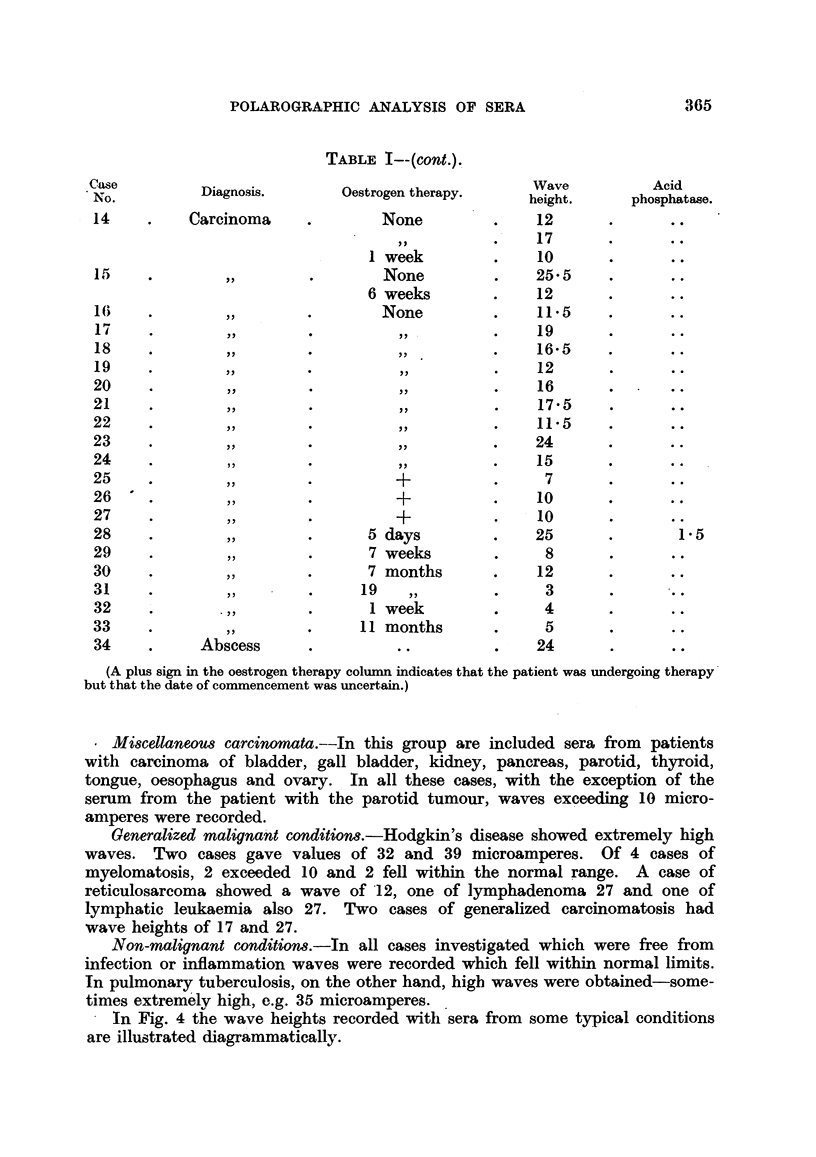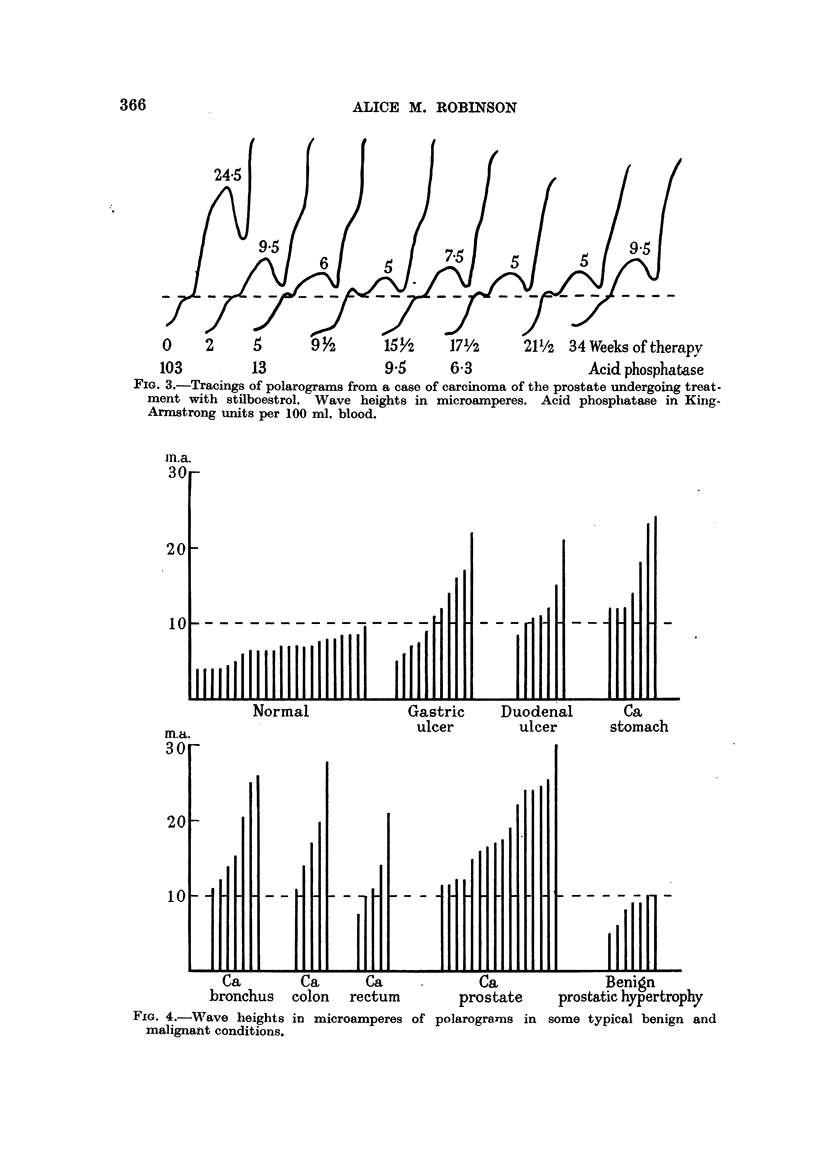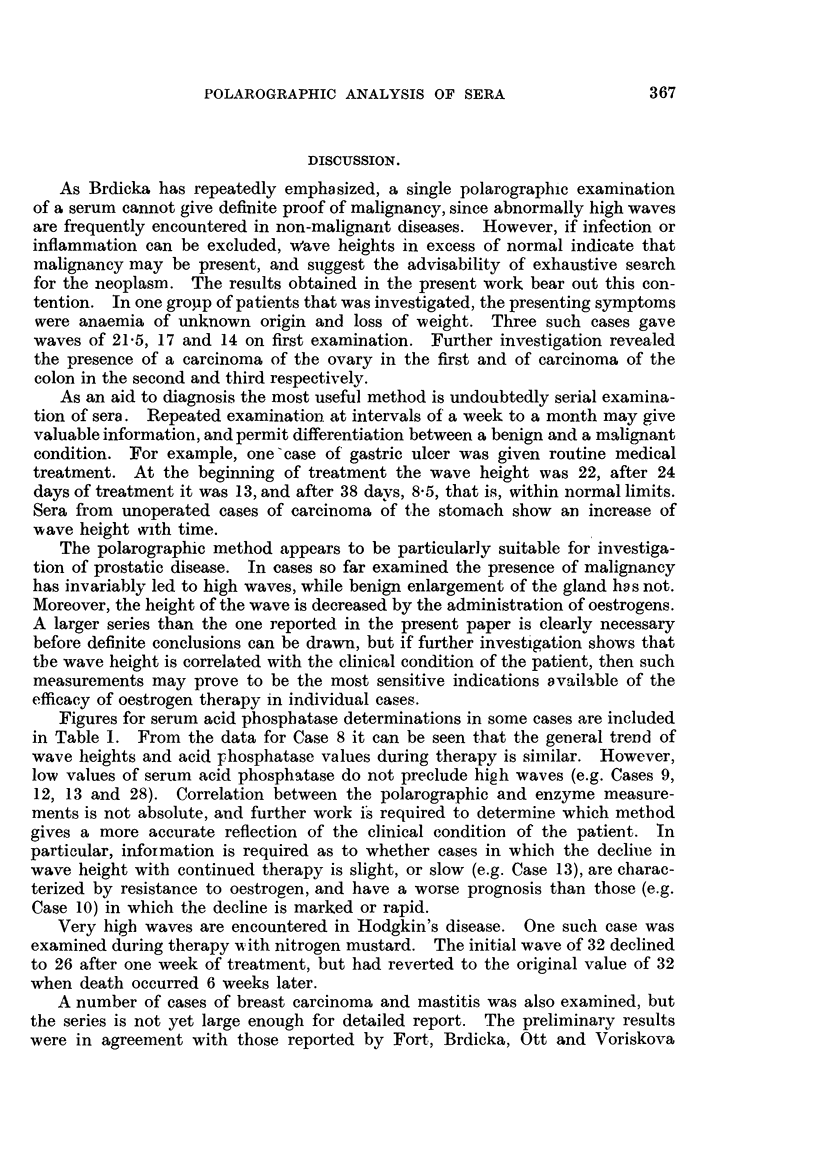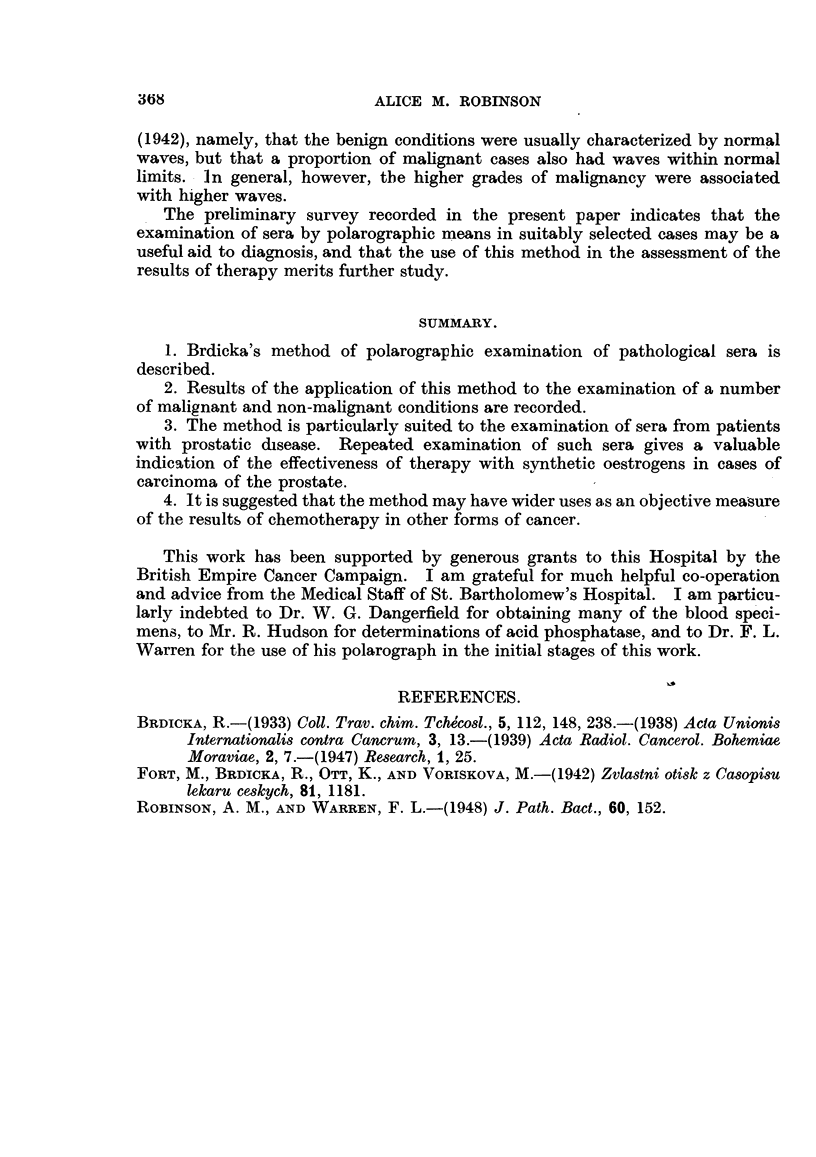# Polarographic Analysis of Normal and Pathological Sera

**DOI:** 10.1038/bjc.1948.39

**Published:** 1948-12

**Authors:** Alice M. Robinson


### POLAROGRAPHIC ANALYSIS OF NORMAL AND PATHOLOGICAL SERA.

ALICE M. ROBINSON.

*From the Pathology Department, St. Bartholomew's Hospital, London, E.C.1.*

Received for publication November 23, 1948.

BRDICKA (1933) discovered that sulphur-containing proteins gave characteristic polarographic waves when examined in ammonia buffer solutions containing traces of cobalt salts. Application of this method of analysis to normal and carcinomatous sera showed that the latter had smaller quantities of sulphur-containing proteins. An important advance in technique occurred when Brdicka (1938; 1939) demonstrated that filtrates prepared from alkali-treated sera after precipitation with sulphosalicylic acid showed much greater differences between normal sera and sera from cancer patients. With the filtrates the difference between the two types of sera was reversed, that is, the sera from cancer patients showed higher waves, indicating the presence of more sulphidic substance in their filtrates.

Further work showed that the presence of a high filtrate wave was not specifically characteristic of cancer sera (Brdicka, 1947). A valuable observation was, however, that in sera from patients undergoing successful treatment (e.g. surgical or irradiation therapy) the abnormally high serum filtrate waves reverted to normal size.

Recent increasing use of chemotherapeutic measures, at least in some forms of malignancy, suggested that the polarographic method might have some value as an objective, albeit purely empirical, measure of the efficacy of such therapy.

With this object in view some preliminary investigations were carried out which have already been briefly reported (Robinson and Warren, 1948). A larger series of sera from normal subjects, from patients with malignant and non-malignant diseases and sera from patients with malignant disease undergoing therapy, has now been studied in an attempt to assess the value of the method and its limitations. The results reported in the present paper are based on the polarographic examination of approximately 300 sera.

### EXPERIMENTAL.

The technique was standardized for use with a Tinsley ink-recording polarograph. The cell was of a simple cylindrical type, open to air. The anode consisted of a pool of mercury of 1–2 cm. diameter. The capillary had the following characteristics: At a pressure of mercury of 71·5 cm. the drop-time was 3·57 seconds (open circuit), and the weight of mercury dropping per second was 1·63 mg. All polarograms were measured at room temperature (15–20° C.) since preliminary observations indicated that the temperature coefficient of the diffusion current was very small.

Blood was collected by venepuncture and allowed to clot. Serum was removed and treated as follows: Serum (0·4 ml.) was diluted with N/10 potassium hydroxide solution (1·0 ml.) and allowed to stand at room temperature for 45 minutes. Proteins were then precipitated by the addition of 20 per cent sulphosalicylic acid solution (1·0 ml.). The tubes containing the precipitated serum were well shaken, and allowed to stand for exactly 10 minutes before filtering through Whatman filter-paper No. 50. The filtrate (0·5 ml.) was added to 5·0 ml. of a buffer of the following composition: N ammonia, N/10 ammonium chloride, M/1000 cobaltihexammine chloride. In order to render the solution oxygen-free, 3 ml. of a freshly-prepared saturated solution of sodium sulphite was incorporated in each 100 ml. of buffer.

Polarograms were run between 0·8 and 1·8 volts with the instrument set at a sensitivity of 50 microamperes. The characteristic waves appeared as rounded maxima having their greatest height at a cathodic potential of about — 1·45 volts. (These waves are different in shape from those shown in publications by Brdicka and his collaborators, since the use of N ammonia instead of the N/10 originally employed by the Czech workers develops rounded maxima instead of the double waves illustrated by them.)

The general nature of the records obtained is illustrated in Fig. 1. Fig. 1 (*b*) shows a current-voltage curve for the cobalt-containing buffer solution alone. The pronounced maximum developed at about — 1·2 volts is produced by the reduction of cobalt ions. The final steep rise in the region of — 1·7 volts corresponds to the discharge of ammonium ions. Fig. 1 (*c*) shows the current-voltage curve for the same buffer solution to which serum filtrate has been added. The maximum on the cobalt curve has been almost entirely suppressed, and the wave for the cobalt ions is now succeeded by a new wave with a peak at approximately — 1·45 volts. The second wave is produced by the interaction of — SH containing molecules and cathodic electrons. The height of this filtrate wave (in microamperes) is taken as the peak height measured from a base line drawn through the point of inflexion between the end of the cobalt wave and the beginning of the filtrate wave. As will be seen in later figures, the actual shape of the current-voltage curve at this point of inflexion is variable but, in practice, there is no difficulty in determining its position with a fair degree of accuracy. The filtrate waves encountered in the present experiments varied in height from a few microamperes (with normal sera) to about 40 microamperes (with pathological sera). With the sensitivity of the instrument set at 50 microamperes (i.e. with the full range of the current axis of the recording chart equivalent to 50 microamperes) all recordings could be made on the same scale, and direct comparisons of sera obtained on different occasions from the same patient facilitated.

### RESULTS.

*Normal sera.*

Sera from 20 normal males gave waves varying in height from about 4 to 10 microamperes, with a mean value of 6·5 microamperes. Repeat observations on the same individual at different times showed little variation. In a series of normal women there was a range of 2 to 8 with a mean of 6·0 microamperes. The type of current-voltage curves obtained with filtrates from normal sera is illustrated in Fig. 2, which shows 8 such curves for normal male sera.

*Pathological sera.*

Most of the sera examined were from patients suspected of cancer. A smaller number of cases on confirmed non-malignant conditions were also investigated for comparison. In the interpretation of polarograms from pathological sera a value of 10 microamperes was taken as representing the upper limit of normality so far encountered in this work. This arbitrary limit was taken for want of a

Fig. 1.—Polarographic tracings.
(a) Ammonia-ammonium chloride buffer.
(b) Buffer plus cobaltihexammine chloride.
(c) Cobaltic buffer plus blood filtrate.

better but, since the wave height for a normal serum can be as low as 2 microamperes in some individuals, values less than 10 microamperes might represent deviations from normality in some cases. However, for the present purpose, all values under 10 are considered as within normal limits.

*Stomach and duodenum.*—Twenty-five cases were examined in which the provisional diagnosis lay between neoplasia or gastric or duodenal ulcer. Seven confirmed cases of carcinoma of the stomach gave waves ranging from 12 to

24 microamperes. Confirmed gastric ulcers (11 cases) gave values from 5 to 22 microamperes, and 6 of these cases lay above the limit of 10 microamperes. Seven cases were confirmed as duodenal ulcers ; 1 serum showed a wave less than 10 microamperes, while 6 ranged up to 21 microamperes.

*Bronchus.*—In 10 suspected cases of carcinoma of the bronchus 8 sera gave wave heights exceeding 10 microamperes. In all but one of these cases the diagnosis was confirmed. The 2 sera giving values below 10 were subsequently shown to be free from malignancy.

*Large intestine and rectum.*—Of 11 cases examined, 5 were later found to have carcinoma of the colon, 5 carcinoma of the rectum and 1 carcinoma of the caecum. Sera from 10 of these cases gave waves greater than 10. One case of rectal carcinoma was 7·5.

FIG. 2.—Tracings of polarograms from filtrates of normal sera. Wave heights are marked in microamperes.

*Prostate.*—Thirty-four cases of prostatic disease were examined. Sera from 7 patients with benign hypertrophy of the gland gave waves varying from 5 to 10 microamperes, that is, all fell within the normal range. All sera from cases of carcinoma showed values in excess of normal, the lowest being 11·5 and the highest 30. One serum from a patient with prostatic abscess had a wave height of 24.

The values just mentioned were obtained on patients not receiving any form of therapy. Most of the carcinoma cases were subsequently treated with synthetic oestrogens—stilboestrol in the majority of cases. Sera from such treated cases were examined at intervals throughout the course of therapy. In general, administration of the oestrogen resulted in a decrease of wave height. The rate and magnitude of this decrease varied in different cases. Usually, under continuous therapy, the wave reverted to normal height in a few weeks. Continued increase in wave height during therapy was taken as an indication for increased dosage of oestrogen. Individual data for these cases, together with some values for serum acid phosphatase determined on the same specimens, are shown in Table I. Tracings of polarograms obtained during therapy of Case 8 are shown in Fig. 3.

TABLE I.—*Wave Heights and Acid Phosphatase Values of Sera from Cases of Prostatic Carcinoma.*

| Case No. | Diagnosis. | Oestrogen therapy. | Wave height. | Acid phosphatase. |
|---|---|---|---|---|
| 1 | Benign | None | 10 | .. |
| 2 | ,, | ,, | 9 | .. |
| 3 | ,, | ,, | 5 | .. |
| 4 | ,, | ,, | 8 | .. |
| 5 | ,, | ,, | 10 | .. |
| 6 | ,, | ,, | 9 | .. |
| 7 | ,, | ,, | 6 | .. |
| 8 | Carcinoma | ,, | 15 | >100 |
| | | ,, | 24·5 | >100 |
| | | 2 weeks | 9·5 | .. |
| | | 5 ,, | 6 | .. |
| | | 9½ ,, | 5 | 17 |
| | | 15½ ,, | 7·5 | 9·5 |
| | | 17½ ,, | 5 | 6·3 |
| | | 21½ ,, | 5 | .. |
| | | 34 ,, | 9·5 | .. |
| 9 | ,, | None | 24 | 1·8 |
| | | 3 weeks | 14 | .. |
| | | 4 ,, | 13 | .. |
| | | 5 ,, | 16 | .. |
| | | 6 ,, | 14 | .. |
| | | 12 ,, | 9 | 1·3 |
| 10 | ,, | None | 22 | .. |
| | | 4 days | 24 | 21 |
| | | 9 ,, | 15 | .. |
| | | 12 ,, | 13 | .. |
| | | 6 weeks | 9 | 1·2 |
| 11 | ,, | 4 months | 8 | 3 |
| | | 5 ,, | 7 | 1·5 |
| | | 6 ,, | 8 | 1·6 |
| | | 10 ,, | 6 | 1·1 |
| 12 | ,, | None | 30 | 6·4 |
| | | 5 days | 18 | .. |
| | | 9 ,, | 14 | .. |
| | | 5 weeks | 9 | 1·2 |
| 13 | ,, | 5 months | 20·5 | 7·5 |
| | | 6 ,, | 16 | 1·6 |
| | | 7 ,, | 10 | 2·5 |
| | | 10 ,, | 15 | 3·6 |

TABLE I—(*cont.*).

| Case No. | Diagnosis. | Oestrogen therapy. | Wave height. | Acid phosphatase. |
|---|---|---|---|---|
| 14 | Carcinoma | None | 12 | .. |
| | | ,, | 17 | .. |
| | | 1 week | 10 | .. |
| 15 | ,, | None | 25·5 | .. |
| | | 6 weeks | 12 | .. |
| 16 | ,, | None | 11·5 | .. |
| 17 | ,, | ,, | 19 | .. |
| 18 | ,, | ,, | 16·5 | .. |
| 19 | ,, | ,, | 12 | .. |
| 20 | ,, | ,, | 16 | .. |
| 21 | ,, | ,, | 17·5 | .. |
| 22 | ,, | ,, | 11·5 | .. |
| 23 | ,, | ,, | 24 | .. |
| 24 | ,, | ,, | 15 | .. |
| 25 | ,, | + | 7 | .. |
| 26 | ,, | + | 10 | .. |
| 27 | ,, | + | 10 | .. |
| 28 | ,, | 5 days | 25 | 1·5 |
| 29 | ,, | 7 weeks | 8 | .. |
| 30 | ,, | 7 months | 12 | .. |
| 31 | ,, | 19 ,, | 3 | .. |
| 32 | ,, | 1 week | 4 | .. |
| 33 | ,, | 11 months | 5 | .. |
| 34 | Abscess | .. | 24 | .. |

(A plus sign in the oestrogen therapy column indicates that the patient was undergoing therapy but that the date of commencement was uncertain.)

*Miscellaneous carcinomata.*—In this group are included sera from patients with carcinoma of bladder, gall bladder, kidney, pancreas, parotid, thyroid, tongue, oesophagus and ovary. In all these cases, with the exception of the serum from the patient with the parotid tumour, waves exceeding 10 microamperes were recorded.

*Generalized malignant conditions.*—Hodgkin's disease showed extremely high waves. Two cases gave values of 32 and 39 microamperes. Of 4 cases of myelomatosis, 2 exceeded 10 and 2 fell within the normal range. A case of reticulosarcoma showed a wave of 12, one of lymphadenoma 27 and one of lymphatic leukaemia also 27. Two cases of generalized carcinomatosis had wave heights of 17 and 27.

*Non-malignant conditions.*—In all cases investigated which were free from infection or inflammation waves were recorded which fell within normal limits. In pulmonary tuberculosis, on the other hand, high waves were obtained—sometimes extremely high, e.g. 35 microamperes.

In Fig. 4 the wave heights recorded with sera from some typical conditions are illustrated diagrammatically.

FIG. 3.—Tracings of polarograms from a case of carcinoma of the prostate undergoing treatment with stilboestrol. Wave heights in microamperes. Acid phosphatase in King-Armstrong units per 100 ml. blood.

FIG. 4.—Wave heights in microamperes of polarograms in some typical benign and malignant conditions.

DISCUSSION.

As Brdicka has repeatedly emphasized, a single polarographic examination of a serum cannot give definite proof of malignancy, since abnormally high waves are frequently encountered in non-malignant diseases. However, if infection or inflammation can be excluded, wave heights in excess of normal indicate that malignancy may be present, and suggest the advisability of exhaustive search for the neoplasm. The results obtained in the present work bear out this contention. In one group of patients that was investigated, the presenting symptoms were anaemia of unknown origin and loss of weight. Three such cases gave waves of 21·5, 17 and 14 on first examination. Further investigation revealed the presence of a carcinoma of the ovary in the first and of carcinoma of the colon in the second and third respectively.

As an aid to diagnosis the most useful method is undoubtedly serial examination of sera. Repeated examination at intervals of a week to a month may give valuable information, and permit differentiation between a benign and a malignant condition. For example, one case of gastric ulcer was given routine medical treatment. At the beginning of treatment the wave height was 22, after 24 days of treatment it was 13, and after 38 days, 8·5, that is, within normal limits. Sera from unoperated cases of carcinoma of the stomach show an increase of wave height with time.

The polarographic method appears to be particularly suitable for investigation of prostatic disease. In cases so far examined the presence of malignancy has invariably led to high waves, while benign enlargement of the gland has not. Moreover, the height of the wave is decreased by the administration of oestrogens. A larger series than the one reported in the present paper is clearly necessary before definite conclusions can be drawn, but if further investigation shows that the wave height is correlated with the clinical condition of the patient, then such measurements may prove to be the most sensitive indications available of the efficacy of oestrogen therapy in individual cases.

Figures for serum acid phosphatase determinations in some cases are included in Table I. From the data for Case 8 it can be seen that the general trend of wave heights and acid phosphatase values during therapy is similar. However, low values of serum acid phosphatase do not preclude high waves (e.g. Cases 9, 12, 13 and 28). Correlation between the polarographic and enzyme measurements is not absolute, and further work is required to determine which method gives a more accurate reflection of the clinical condition of the patient. In particular, information is required as to whether cases in which the decline in wave height with continued therapy is slight, or slow (e.g. Case 13), are characterized by resistance to oestrogen, and have a worse prognosis than those (e.g. Case 10) in which the decline is marked or rapid.

Very high waves are encountered in Hodgkin's disease. One such case was examined during therapy with nitrogen mustard. The initial wave of 32 declined to 26 after one week of treatment, but had reverted to the original value of 32 when death occurred 6 weeks later.

A number of cases of breast carcinoma and mastitis was also examined, but the series is not yet large enough for detailed report. The preliminary results were in agreement with those reported by Fort, Brdicka, Ott and Voriskova

(1942), namely, that the benign conditions were usually characterized by normal waves, but that a proportion of malignant cases also had waves within normal limits. In general, however, the higher grades of malignancy were associated with higher waves.

The preliminary survey recorded in the present paper indicates that the examination of sera by polarographic means in suitably selected cases may be a useful aid to diagnosis, and that the use of this method in the assessment of the results of therapy merits further study.

### SUMMARY.

1. Brdicka's method of polarographic examination of pathological sera is described.
2. Results of the application of this method to the examination of a number of malignant and non-malignant conditions are recorded.
3. The method is particularly suited to the examination of sera from patients with prostatic disease. Repeated examination of such sera gives a valuable indication of the effectiveness of therapy with synthetic oestrogens in cases of carcinoma of the prostate.
4. It is suggested that the method may have wider uses as an objective measure of the results of chemotherapy in other forms of cancer.

This work has been supported by generous grants to this Hospital by the British Empire Cancer Campaign. I am grateful for much helpful co-operation and advice from the Medical Staff of St. Bartholomew's Hospital. I am particularly indebted to Dr. W. G. Dangerfield for obtaining many of the blood specimens, to Mr. R. Hudson for determinations of acid phosphatase, and to Dr. F. L. Warren for the use of his polarograph in the initial stages of this work.

### REFERENCES.

BRDICKA, R.—(1933) *Coll. Trav. chim. Tchécosl.*, **5**, 112, 148, 238.—(1938) *Acta Unionis Internationalis contra Cancrum*, **3**, 13.—(1939) *Acta Radiol. Cancerol. Bohemiae Moraviae*, **2**, 7.—(1947) *Research*, **1**, 25.

FORT, M., BRDICKA, R., OTT, K., AND VORISKOVA, M.—(1942) *Zvlastni otisk z Casopisu lekaru ceskych*, **81**, 1181.

ROBINSON, A. M., AND WARREN, F. L.—(1948) *J. Path. Bact.*, **60**, 152.